# Cholesterol nose-to-brain delivery as a possible therapeutic strategy in Huntington’s disease

**DOI:** 10.1186/s40035-026-00569-x

**Published:** 2026-08-04

**Authors:** Monica Favagrossa, Alice Passoni, Marta Valenza, Daria Di Prisco, Giulia Birolini, Michela Villa, Andrea Scolz, Laura Pasetto, Valentina Bonetto, Chiara Zuccato, Caterina Mariotti, Renzo Bagnati, Elena Cattaneo, Laura Colombo, Mario Salmona

**Affiliations:** 1 Department of Molecular Biochemistry and Pharmacology, Istituto Di Ricerche Farmacologiche Mario Negri IRCCS, Milan, Italy; 2 Istituto Nazionale Di Genetica Molecolare “Romeo Ed Enrica Invernizzi”, Milan, Italy; 3 Fondazione IRCCS Istituto Neurologico Carlo Besta, Milan, Italy; 4 Department of Environmental Health Science, Istituto di Ricerche Farmacologiche Mario Negri IRCCS, Milan, Italy; 5 Department of Biosciences, University of Milan, Milan, Italy; 6 Department of Neuroscience, Istituto di Ricerche Farmacologiche Mario Negri IRCCS, Milan, Italy

**Keywords:** Huntington’s disease, Cholesterol, Intranasal administration, Liposomes, Nose-to-brain delivery

## Abstract

**Background:**

Huntington’s disease (HD) is a genetically dominant neurodegenerative disorder characterized by several pathological mechanisms, including the disruption of brain cholesterol homeostasis. In several HD animal models, brain cholesterol biosynthesis and levels are reduced. Since circulating cholesterol cannot reach the brain, providing exogenous cholesterol has been shown to improve HD phenotypes. However, the methods used for cholesterol delivery were invasive and not easily transferable to clinical practice.

**Methods:**

Cholesterol-enriched liposomes were developed by using freeze-and-thaw methods and were administered to R6/2 mice through a single or repeated intranasal administrations. Deuterated-cholesterol was used to discriminate exogenous from endogenous cholesterol. Exogenous cholesterol accumulation and distribution, as well as the levels of cholesterol precursors and metabolites, were measured using mass spectrometry. Behavioral tests, real-time PCR analysis, and immunostaining of mutant HTT (muHTT) aggregates were performed to verify the therapeutic effects of liposomes. Plasma neurofilament levels were measured by Simoa-Quanterix assay.

**Results:**

We developed and characterized freeze-and-thaw liposomes. Then, we demonstrate that the exogenous cholesterol can spread throughout the entire brain following intranasal administration of cholesterol-enriched liposomes. Furthermore, repeated intranasal treatments with liposomes result in a full restoration of cognitive decline, and delayed the onset of coordination and motor impairment as well as the loss of muscular strength in the early stages of the disease. Cholesterol supplementation also reduced the plasma level of neurofilament light chain and promoted the clearance of muHTT aggregates.

**Conclusions:**

The findings support the effectiveness of cholesterol supplementation as a therapeutic strategy for HD and indicate the translational potential of nose-to-brain cholesterol delivery.

**Supplementary Information:**

The online version contains supplementary material available at 10.1186/s40035-026-00569-x.

## Introduction

Huntington’s disease (HD) is a genetically dominant neurodegenerative disorder that leads to progressive cognitive and motor decline and poses serious challenges for patients, families, and healthcare providers. In response to these challenges, researchers are working to understand disease mechanisms and develop new therapies. One area that has garnered significant attention is the role of cholesterol in brain function and its potential implication in HD pathogenesis. As a cholesterol-rich organ, the brain relies heavily on this crucial molecule to maintain its structure and function. Cholesterol is a vital component of myelin sheaths and cell membranes and plays a pivotal role in signal transduction and overall neuronal health. Because the blood–brain barrier (BBB) prevents cellular uptake of lipids from the bloodstream, the brain’s need for cholesterol is met by de novo cholesterol synthesis [1].

Interestingly, extensive research has identified significant impairments in cholesterol biosynthesis within the brain in various HD animal models, leading to reduced brain cholesterol levels in later disease stages. Notably, cholesterol biosynthesis is also affected in the presymptomatic stage of the disease, suggesting that cholesterol dysregulation may be an early event in HD pathogenesis [2–7]. The discovery of the potential role of cholesterol in HD has led to the development of two distinct therapeutic approaches. The first is based on enhancing cholesterol availability in the brain [8–13], and the second relies on promoting neuronal cholesterol catabolism through forced expression of the neuronal enzyme, cholesterol 24-hydroxylase (CYP46A) [14, 15]. Although these two cholesterol-based approaches may appear opposite, they converge on the same therapeutic goal: the restoration of cholesterol metabolism, which is consistently beneficial across different HD mouse models, underscoring their therapeutic potential.

Here, we followed the first approach, but a major challenge lies in delivering cholesterol effectively to the brain. While crucial for maintaining the brain’s homeostasis, the BBB presents a significant obstacle to drug delivery to the central nervous system (CNS). Most peripherally administered drugs, such as those delivered via intraperitoneal injection, have limited access to the CNS and often undergo metabolism and clearance before reaching their target, thus diminishing their efficacy. This limitation has spurred the search for alternative delivery methods to bypass the BBB and directly target the brain. Given these challenges, we explored non-invasive strategies to deliver cholesterol to the CNS. Among these strategies, intranasal delivery has emerged as a promising approach. The neuroepithelium of the olfactory region provides a unique interface between the peripheral environment and the brain and so offers a potential route for drug delivery that bypasses the BBB. Drug transport from the nasal cavity to the CNS can occur through multiple pathways, including the olfactory and trigeminal nerve pathways and other routes involving vasculature, cerebrospinal fluid and the lymphatic system [16, 17]. Therefore, drugs for nose-to-brain delivery must have appropriate viscosity, physiological tonicity, and pH compatible with the nasal mucosa [17–19]. Different strategies have been studied. Particularly, nanoparticulate approaches, such as liposomes, have demonstrated a high capacity to overcome the challenges of the intranasal route. These approaches allow drug accumulation in the brain while avoiding systemic side effects. In recent years, liposomes have been utilized as carriers for vaccines, drugs, and tracers for imaging, as well as for administering molecules used in diagnosing and treating neurological diseases [20].

Here, we investigate the potential of intranasal delivery of liposomes, composed of cholesterol and phosphatidylcholine, as a non-invasive method for delivering cholesterol into the brains of HD mice. The positive effects observed across multiple domains—cognitive function, motor abilities, and neuropathological markers—strengthen the rationale for translating cholesterol treatment to clinical applications, with this delivery system offering a promising approach.

## Materials and methods

### Freeze-and-thaw liposomes and characterization

Cholesterol was entrapped in liposomes following the freeze-and-thaw method. Briefly, cholesterol and phosphatidylcholine were dissolved in chloroform at a 1:1 molar ratio and deposited as a dry lipid film in a round-bottomed glass tube following the organic solvent’s evaporation under a nitrogen stream. The lipid film was put in a vacuum desiccator for 30 min to remove organic solvent residue and then rehydrated with phosphate buffer solution (PBS, pH 7.4) to a concentration of 14.4 mM. The lipids were dipped into liquid nitrogen for rapid cooling. After 30 s, the frozen lipids were transferred to a bath at 60 °C for thawing. This operation was repeated several times. Liposomes were then sonicated for 1 min and diluted to a final cholesterol concentration of 5.6 mg/mL.

The characterization of the liposomes was performed using dynamic light scattering and ζ-potential analysis [21]. Measurements were carried out using a Zetasizer Nano ZS (Malvern Panalytical, Worcestershire, UK) at 25 °C. For dynamic light scattering analysis, liposome samples were diluted 1:100 in Dulbecco's Phosphate Buffered Saline and analyzed to determine the hydrodynamic diameter and polydispersity index. Each sample was measured in triplicate, and the mean diameter was reported. The ζ-potential was determined using the same instrument by applying electrophoretic light scattering. To assess colloidal stability, liposome suspensions were stored at room temperature and analyzed at defined time points (0, 2, 24, and 48 h, and 1 week). Changes in hydrodynamic diameter and ζ-potential over time were monitored to evaluate size and surface charge stability.

### Animal model

Procedures involving animals and their care were conducted in accordance with the institutional guidelines at the Istituto di Ricerche Farmacologiche Mario Negri IRCCS and in compliance with national (D.lgs 26/2014; Authorization n. 19/2008-A issued March 6, 2008, by the Ministry of Health) and international laws and policies (EEC Council Directive 2010/63/U.E.; NIH Guide for the Care and Use of Laboratory Animals, 2011 edition). Animal procedures were reviewed and approved by the Mario Negri Institute Animal Care and Use Committee, which includes ad hoc members for ethical issues, and by the Italian Ministry of Health (authorization numbers 616/2018-PR and 786/2021-PR, response to protocols 9F5F5.117 and 9F5F5.199). Animal facilities meet international standards and are regularly checked by a certified veterinarian who is responsible for monitoring health, supervising animal welfare and reviewing experimental protocols.

We used an R6/2 colony generated to overexpress the first exon of the human mutant huntingtin gene with approximately 160 CAG repeats, causing the suffering phenotypes that characterize R6/2 mice, regardless of sex. The animals were kept on a 12-h night/day cycle with access to water and food ad libitum. Our R6/2 colony was maintained by crossing heterozygous R6/2 males and commercial wild-type (WT) females (B6CBAF1/J, purchased from Charles River). The R6/2 line was genotyped by polymerase chain reaction (PCR) on DNA from ear biopsy tissue at weaning [22]. The experiments were carried out on R6/2 mice and their WT littermates.

### Mouse treatments

Cholesterol-enriched liposomes were administered intranasally immediately after their preparation. Each mouse received administration of 6 µL of a 5.6 mg/mL liposome suspension at the inner surface of each nostril. The administration was repeated three times, resulting in a total of 36 µL of liposome suspension, corresponding to a final dose of 200 µg of intranasal cholesterol in each mouse. After each intranasal administration, a treat was given as positive reinforcement [23].

For the pharmacokinetic study, 6-week-old R6/2 and WT mice (*n* = 3–5 per genotype per time point) received a single intranasal (IN) administration. At 6, 24, 48, 72, 96, 144, 192, or 240 h post-treatment, animals were deeply anesthetized via intraperitoneal injection of an overdose of ketamine hydrochloride (IMALGENE, 150 mg/kg; #ALC104135013, Alcyon Italia) and medetomidine hydrochloride (DOMITOR, 2 mg/kg; #ALC100103011, Alcyon Italia), and subsequently euthanized for immediate tissue collection.

For sub-chronic experiments, each mouse received one intranasal administration of liposomes or PBS (vehicle) per week, from 5 to 11 weeks of age, for a total of 7 administrations.

At sacrifice, blood samples were collected with the anticoagulant K_3_EDTA, and plasma samples were obtained by centrifugation at 5000×*g* for 5 min. Peripheral tissues (liver and kidney) and brains were immediately removed; the bulbs, striatum, cortex, cerebellum and hippocampus were collected separately. All samples were stored at − 80 °C until analysis.

### Behavioral tests

Mouse behavior during sub-chronic treatment was evaluated at specific time points. Considering that both male and female animals were included in each behavioral test, and we have not determined any sex effect, we decided to sum up male and female performances in behavioral test results. Mice with epileptic seizures were excluded from all the tests performed.

#### Rotarod test

Mice were tested over three consecutive days at 8 and 10 weeks of age. First, animals were trained on a rotating bar at 4 rpm for 5 min (apparatus model 47600, Ugo Basile, Milan, Italy). One hour later, mice were tested in three consecutive trials of 5 min, with the rotarod speed linearly increasing from 4 to 40 rpm in each trial. The latency to fall from the rod was recorded for each trial and averaged.

#### Novel object recognition test (NORT)

Mice were tested at 9 and 11 weeks. In the habituation stage, each mouse was placed into an empty non-reflective arena (40 × 40 × 40 cm^3^) for 10 min. In the familiarization stage, two identical objects (A and A′) were presented to each animal for 10 min. In the test stage on the next day, animals were exposed to one familiar object (A) and a new object (B) for 10 min. All phases of the test were conducted with a low-intensity white light source. The discrimination index was calculated as (time exploring the novel object – time exploring the familiar object)/(total time exploring the two objects). Mice exploring less than 7 s were excluded from the analysis due to their inability to perform the task.

#### Grip strength test

Mice were tested at 8 and 10 weeks of age. The animals were lifted by the tail, lowered toward the grip (apparatus model GT3, Bioseb, Vitrolles, France), and then gently pulled straight back with consistent force until they released the grip. The forelimb grip force, measured in grams, was recorded. The test was repeated five times, and the measurements were averaged.

### Sample preparation for deuterated-cholesterol (chol-D6) determination

The chol-D6 levels were determined with a previously published and validated method, using a 1200 Series HPLC system (Agilent Technologies, Santa Clara, CA) interfaced to an API 5500 triple quadrupole mass spectrometer (Sciex, Thornhill, Canada) [9]. Briefly, plasma samples (50 µL) were diluted with 200 µL of ethanol spiked with β-sitosterol, used as internal standard. Brain samples (40 mg) were homogenized with 1 mL of ethanol/water 4:1 (*v*/*v*), containing 500 ng of internal standard. The supernatants were centrifugated for 15 min at 4 °C, and 4 µL was injected directly into the liquid chromatography-mass spectrometry (LC–MS) system. Chol-D6 and β-sitosterol were separated on a Gemini C18 column (50 × 2 mm^2^; 5 μm particle size), using an isocratic gradient in methanol at 35 °C.

The mass spectrometer was equipped with an atmospheric pressure chemical ionization source operating in positive ion and selected reaction monitoring mode to measure the product ions obtained in a collision cell from the [M–H_2_O]^+^ ions of the analytes (Table S1).

### Imaging mass spectrometry (IMS)

#### Sample preparation

Frozen brains were cut into 10-µm-thick sections using a cryo-microtome (Leica Microsystems, Wetzler, Germany) at − 20 °C. Three coronal sections were cut from the whole brains of R6/2 mice at 12 weeks and mounted on a – 20 °C pre-cooled MALDI plate by standard thaw-mounting techniques (gently touching the back of the frozen MALDI plate with a finger) and stored at − 80 °C until further analysis. The plate with the tissue sections was dried in a vacuum at room temperature for 1 h. A solution of chol-D6 at 200 ng/µL, used as internal standard, was sprayed from a SunCollect automated pneumatic sprayer (SunChrom supplied by K.R. Analytical Ltd., UK) using the following parameters: flow rate 20 μL/min, linear velocity 900 mm/min, 2 mm line distance, Z position 30 mm, 54 layers. As an internal standard, we chose a cholesterol-D6 molecule, deuterated on the aliphatic chain rather than on the steroid rings (Sigma-Aldrich, #679046, Burlington, MA), to avoid structural modification of the deuterated portion during the derivatisation reaction (Fig. S2a).

#### Sample derivatization

We adapted a strategy from Angelini et al. for mapping the cholesterol distribution in brain slices [24]. Briefly, the sprayer was thoroughly flushed with ~ 2 mL methanol, after which cholesterol oxidase (0.264 units/mL in 5 mM KH_2_PO_4_, pH 7) was sprayed in 18 layers. The first layer was applied at 10 μL/min, the second at 15 μL/min, and then all subsequent layers at 20 μL/min to give an enzyme density of 0.05 units/mm^2^. After that, the enzyme-coated slide was placed on a flat dry bed above 30 mL of warm water (37 °C) in a closed pipette-tip box (11.9 × 8.2 × 8.5 cm^3^) and then incubated at 37 °C for 1 h, after which the slide was removed and dried in a vacuum desiccator for 15 min.

Girard’s T reagent (GT) (6.3 mg/mL bromide salt in 70% methanol with 5% acetic acid) was sprayed on the dried slide with the same spray parameters used for spraying cholesterol oxidase. The resulting GT density was 1.21 μg/mm^2^. The slide was then placed on a flat dry bed above 30 mL of pre-warmed (37 °C) 50% methanol and 5% acetic acid in a covered glass chamber (12 × 12 × 7.2 cm^3^) and incubated in a water bath at 37 °C for 1 h. The slide was removed and dried in a vacuum desiccator until IMS analysis.

#### Instrumental analysis

A Q Exactive Hybrid Quadrupole-Orbitrap mass spectrometer (Thermo Fisher Scientific, Waltham, MA) was used for IMS experiments, coupled with an AP-MALDI-ng-UHR ion source (MassTech Inc., Columbia, MD), controlled by Target-ng software (MassTech Inc.). Tune software (Thermo Fisher Scientific) was used to control the mass spectrometer parameters and to manage the MS acquisition. Parameters used, unless otherwise specified, were as follows: laser energy, 12.5%; voltage applied to the plate, 2 kV; capillary temperature, 250 °C; and S-lens RF, 80%. In preliminary experiments, each metabolite was co-spotted, after derivatization, with the matrix on the plate and acquired using spiral motion to study the ionization and the fragmentation pattern of each. For IMS experiments, constant speed raster motion was used, with 100 μm spatial resolution and plate velocity dependent on the scan time. Acquisitions were performed in full scan with a positive polarity, a mass range of m/z 100–1000, and a resolution of 70,000 at 200 m/z. Automatic gain control (AGC) was set at 5e^+6^, with 150 ms maximum injection time. The chosen AGC value was high enough to guarantee that all scans reached a maximum injection time, ensuring equal scan times for each pixel.

Data were exported as imzML files from Image Quest (Thermo Fisher Scientific) and imported into MSiReader v1.20. The metabolite ion signal (tolerance ± 5 ppm) was normalized in each pixel to the signal of the internal standard. Quantitative analysis of IMS experiments was carried out as previously described by Falcetta et al. [25]. Calibration curves were generated to convert pixel intensity into cholesterol amounts (ng/mm^2^). Regions of interest (ROIs) were manually selected, and signal intensity was normalized to chol-D6 concentration per mm^2^ in each ROI. The average cholesterol concentration in tissue sections (ng/mm^2^) was calculated by applying the calibration curve to the two-dimensional ROI. Section thickness (10 µm, constant across all sections) was recorded only as a reference parameter to ensure comparability between tissue sections and was not used in the calculation of areal density. IMS provides local surface cholesterol densities (ng/mm^2^), including both endogenous and exogenous cholesterol within the imaged region.

### Sample preparation for analysis of cholesterol precursors and metabolites

The plasma and brain levels of cholesterol precursors and oxysterols were analyzed using a recently published and validated method [26]. All the Multiple-Reaction Monitoring (MRM) transitions, including quantifier and qualifier, are listed in Table S2.

Plasma samples were spiked with internal standard and deproteinized by ethanol using a slow protein precipitation method. The supernatant was then hydrolyzed with 1 M KOH in water, and the reaction was carried out under controlled temperature conditions at 37 °C for 1 h. The samples were then added to 500 µL of water and 2 mL of dichloromethane (DCM) for liquid–liquid extraction. The extraction was repeated twice, with the organic phase recovered and gently dried under nitrogen flow. The extracts were resuspended in 40 µL ethanol for the instrumental analysis.

Brain tissues were homogenated at a 1:10 ratio in PBS solution after adding internal standard. A TeSeE PRECESS 24 system (Bio-Rad, Hercules, CA) was used for tissue homogenization; it uses a three-dimensional rotation movement to fully homogenate tissues. The homogenates were transferred to 1.5 mL tubes and extracted with 800 μL of ethanol containing 100 µg/mL butylated hydroxytoluene. The samples were vortexed and centrifuged at 13,200 rpm for 15 min at 4 °C. The supernatant was then recovered and hydrolyzed with 125 µL of 1 M KOH in water. The hydrolyzed samples were added to 500 µL of water and 2 mL of DCM for liquid–liquid extraction. The extraction was repeated twice, with the organic phase recovered and dried under nitrogen flow. The extracts were resuspended in 40 µL of ethanol for the instrumental analysis.

### Plasma neurofilament analysis

Murine blood samples were processed at sacrifice, and plasma samples were aliquoted and stored at − 80 °C until biomarker analyses. Levels of neurofilament light chain (NfL) were determined using a commercially available single-molecule array assay kit (Quanterix Corp, NF-LIGHT V2 kit, Boston, MA) on an SR-X Analyzer (Quanterix).

### Immunofluorescence analysis

Mice were deeply anesthetized by intraperitoneal (i.p.) injection of a cocktail of ketamine/medetomidine and transcardially perfused with PBS followed by 4% paraformaldehyde. Brains were post-fixed for 2 h in the same solution at 4 °C and then in 30% sucrose to prevent ice crystal damage during freezing in OCT. Immunofluorescence was performed on 10-μm coronal sections. Epitopes were demasked at 98 °C with sodium citrate 10 mM, and then slices were incubated with mouse anti-huntingtin clone EM48 (1:100, #MAB5374, Millipore, Burlington, MA) for 3 h at room temperature (RT). Anti-mouse Alexa Fluor 568‐conjugated goat secondary antibody (1:500; #A-11004, Invitrogen, Waltham, MA), used for detection, was incubated for 1 h at RT. Sections were counterstained with the nuclear dye Hoechst 33258 (1:10,000, #911004-45-0, Invitrogen) and then mounted under coverslips using Vectashield mounting medium (Vector Laboratories, #H-1000-10, Newark, CA). The EM48 antibody specifically targets the expanded polyQ tract prone to aggregation; therefore, the analysis was not conducted on WT mice, as they do not possess the expanded polyQ tract.

To count muHTT aggregates, confocal images were acquired with a LEICA SP5 laser scanning confocal microscope (Leica Microsystems Srl, Milan, Italy). Laser intensity and detector gain were maintained constant for all images, and images were acquired at 40× . To count aggregates in the brains of mice treated with liposomes, 18 images/mouse throughout the striatum, cortex, hippocampus, and olfactory bulbs were taken. To quantify the number and the size of aggregates, ImageJ software (“Analyze Particles”) was used to measure the fluorescence. Images were divided into two-color channels, and the same global threshold was set.

### Gene expression analysis

Following sub-chronic intranasal treatment, total RNA was isolated from striatum tissues from mice using Trizol reagent (Invitrogen, #15596026) and a Pure Link RNA Mini Kit (Invitrogen, #12183018A) according to the manufacturer’s instructions. The quality of the extracted RNA was checked by a NanoDrop ND-1000 (Thermo Fisher Scientific). The genomic DNA present in the extracted RNA samples was digested using the DNase I amplification grade kit (Invitrogen, #18068-015). Then 1000 ng of RNA was reverse transcribed into cDNA using the High-Capacity cDNA Reverse Transcription Kit (Applied Biosystem, #4368813, Waltham, MA). To confirm regional identities, qPCR was performed using primers specific to each brain region: *Drd2* (striatum), *Tbr1* (cortex), and *Shox2* (thalamus). Striatal and cortical markers served as positive controls, while the thalamic marker served as a negative control (data not shown).

Gene expression was evaluated with the QuantStudio 5 Real-Time PCR System (Applied Biosystems). Reactions were performed using PowerSYBR Green PCR Master Mix (Applied Biosystems, #4368577) in the presence of 0.5 µM forward and reverse primers. The amounts of target transcripts were normalized to β-actin as a reference gene. The primer sequences are shown in Table S3.

### Statistical analysis

G-power analysis was used based on pilot or previous studies to predetermine sample sizes. Before data analysis, Grubbs’ test was applied to determine the presence of outliers. GraphPad Prism 8 was used for data analysis. For each set of data to be compared, we determined whether data were normally distributed to select parametric or nonparametric statistical tests. Data are presented as means ± standard deviation. Specific statistical analysis methods are provided in figure legends. *P* < 0.05 indicates statistical significance. Table S4 summarizes all the trials and read-outs performed.

## Results

### Single intranasal administration of cholesterol-enriched liposomes in WT and R6/2 mice

**Fig. 1:**
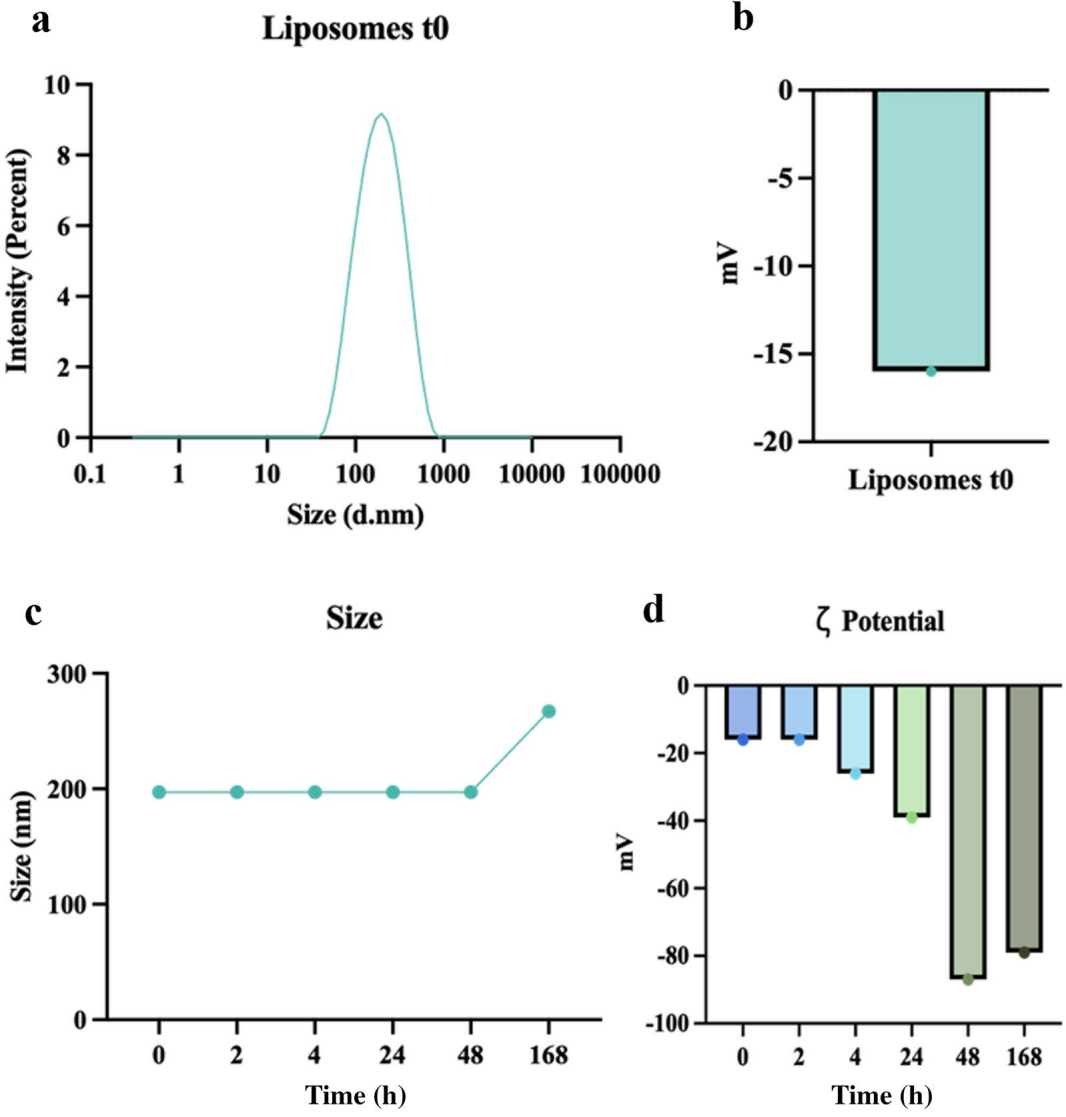
Characterization of chol-D6-enriched liposomes. **a** Dynamic light scattering analysis showed a hydrodynamic diameter of approximately 197 nm. **b** A negative ζ-potential of − 16 ± 0.8 mV was also measured. **c** The size stability test indicated that the nanoparticles remained stable up to 48 h. **d** The ζ-potential showed stability only up to 2 h, with a significant surface charge change observed after one week

In a previous study, we demonstrated that exogenous cholesterol reached various brain regions after intranasal administration of cholesterol-enriched liposomes in WT mice, persisting till 72 h post-treatment [9]. In this study, we first characterized cholesterol-enriched liposomes prepared using the freeze-and-thaw method. Dynamic light scattering analysis (Fig. 1a) indicated that the liposomes had a hydrodynamic diameter of approximately 197 nm. The surface charge, assessed through ζ-potential measurements, was found to be negative, with a value of − 16 ± 0.8 mV (Fig. 1b), consistent with the liposomal composition based on cholesterol. Stability tests revealed that the particle size remained consistent for up to 48 h (Fig. 1c), whereas the ζ-potential was stable only for 2 h. Notably, a marked alteration in surface charge was observed after one week, highlighting a difference in stability between size and surface charge (Fig. 1d).

The absorption and release kinetics of exogenous cholesterol and its concentration over an extended time (up to 240 h) were evaluated in WT and R6/2 mice following a single intranasal administration. The animals were treated with liposomes composed of chol-D6. This enabled measurement of the exogenous cholesterol levels in various tissues by mass spectrometry [9, 12].

**Fig. 2:**
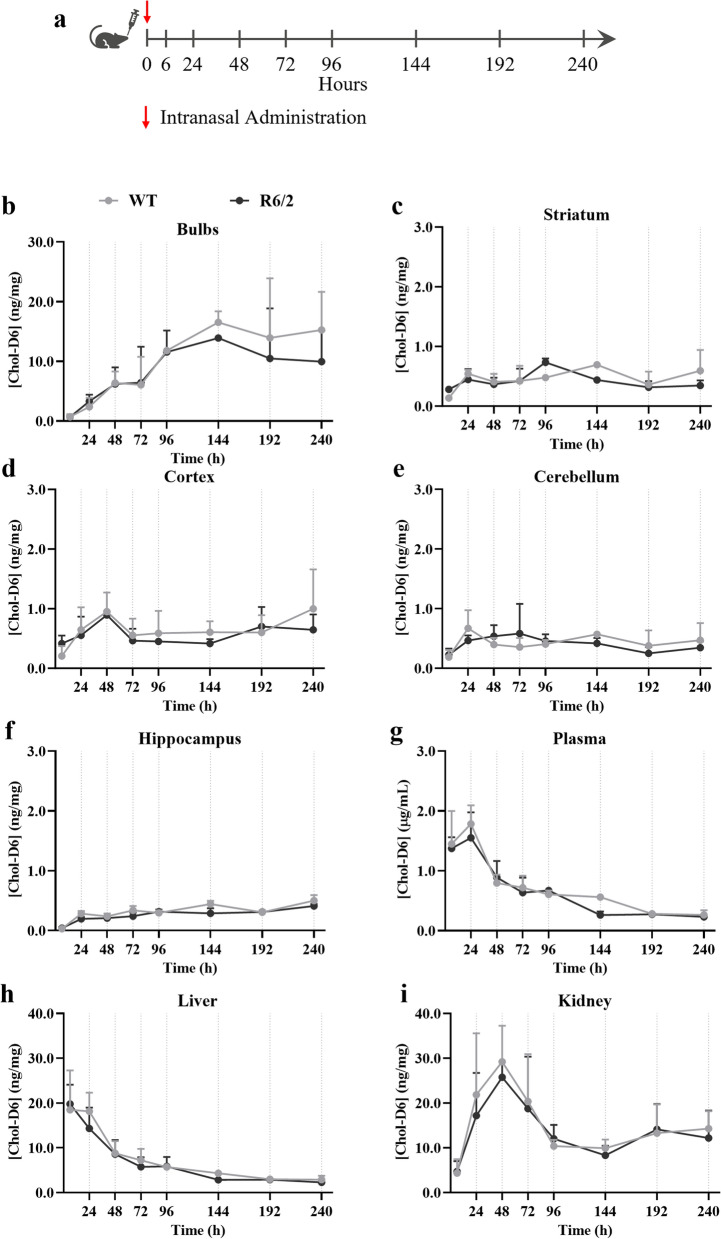
Pharmacokinetic profiles of cholesterol-D6 in WT and R6/2 mice following a single intranasal administration. **a** WT and R6/2 mice were treated with a single dose of chol-D6-enriched liposome (200 µg chol-D6) and sacrificed at different time points. **b-i** Different brain areas, peripheral tissues, and plasma were collected for mass spectrometry analysis (*n* = 3–7 mice/genotype at each time point). Levels of chol-D6 in** b** bulbs, **c** striatum, **d** cortex,** e** cerebellum,** f** hippocampus, **g** plasma, **h** liver and **i** kidney were measured by LC–MS analysis. Data are shown as mean ± standard deviation. Statistics: two-way ANOVA test with a post-hoc Tukey test

All animals received chol-D6-enriched liposomes (200 µg chol-D6) through a single intranasal administration and were sacrificed at 6, 24, 48, 72, 96, 144, 192, or 240 h post-treatment (Fig. 2a). In all tissues analyzed, chol-D6 exhibited a similar kinetic profile in both genotypes for up to 240 h post-treatment (Fig. 2b–i). In brain regions including the striatum, cortex, cerebellum and hippocampus, chol-D6 level increased until 24 h and then was stabilized up to 240 h. These results confirmed findings from our previous study on WT animals [9] and expanded knowledge about cholesterol kinetics over an extended time (Fig. 2c − e). Similarly, in the olfactory bulbs, chol-D6 levels increased, reaching their highest concentrations at later time points, likely due to the proximity of the bulbs to the nasal cavity, the site of intranasal administration (Fig. 2b).

The concentration of chol-D6 remained high in kidney at 240 h after treatment, suggesting that excess exogenous cholesterol was continuously released from tissues and excreted via the kidneys (Fig. 2i). In contrast, the kinetic profiles of chol-D6 in plasma and liver exhibited a typical pattern: a peak in early hours post-administration followed by a progressive decrease till 240 h (Fig. 2g, h).

These results corroborate our previous findings and demonstrate that chol-D6 could effectively cross the BBB and distribute uniformly throughout key brain regions, including the cerebral cortex and striatum, following intranasal administration [9]*.* Furthermore, chol-D6 remained in the brain till at least 240 h post-treatment, suggesting its incorporation into endogenous cholesterol homeostasis. Notably, the pharmacokinetic profiles were similar between WT and R6/2 mice, confirming no significant genotype-dependent differences (Fig. 2).

### Sub-chronic intranasal cholesterol-enriched liposomes in WT and R6/2 mice

We conducted a pilot study on WT mice to assess the safety of sub-chronic liposome administration concerning animal welfare. Based on the results from the single intranasal treatment, where chol-D6 reached a steady-state concentration until 240 h (10 days), we decided to treat animals once a week from 5 to 11 weeks of age. One group of WT mice received liposomes enriched with chol-D6 (200 μg per intranasal administration), while another received PBS as the control. Analysis showed that chol-D6 accumulated in the brains of WT mice after repeated treatments, distributed evenly across different brain regions without accumulation in peripheral tissues (Fig. S1a). Importantly, there were no adverse effects on animal body weight, or cognitive function and motor ability tested with the NORT and rotarod test, respectively (Fig. S1b-d). This initial pilot study demonstrated the safety of this approach, as no adverse effects on animal welfare were observed in WT mice undergoing sub-chronic treatment.

**Fig. 3:**
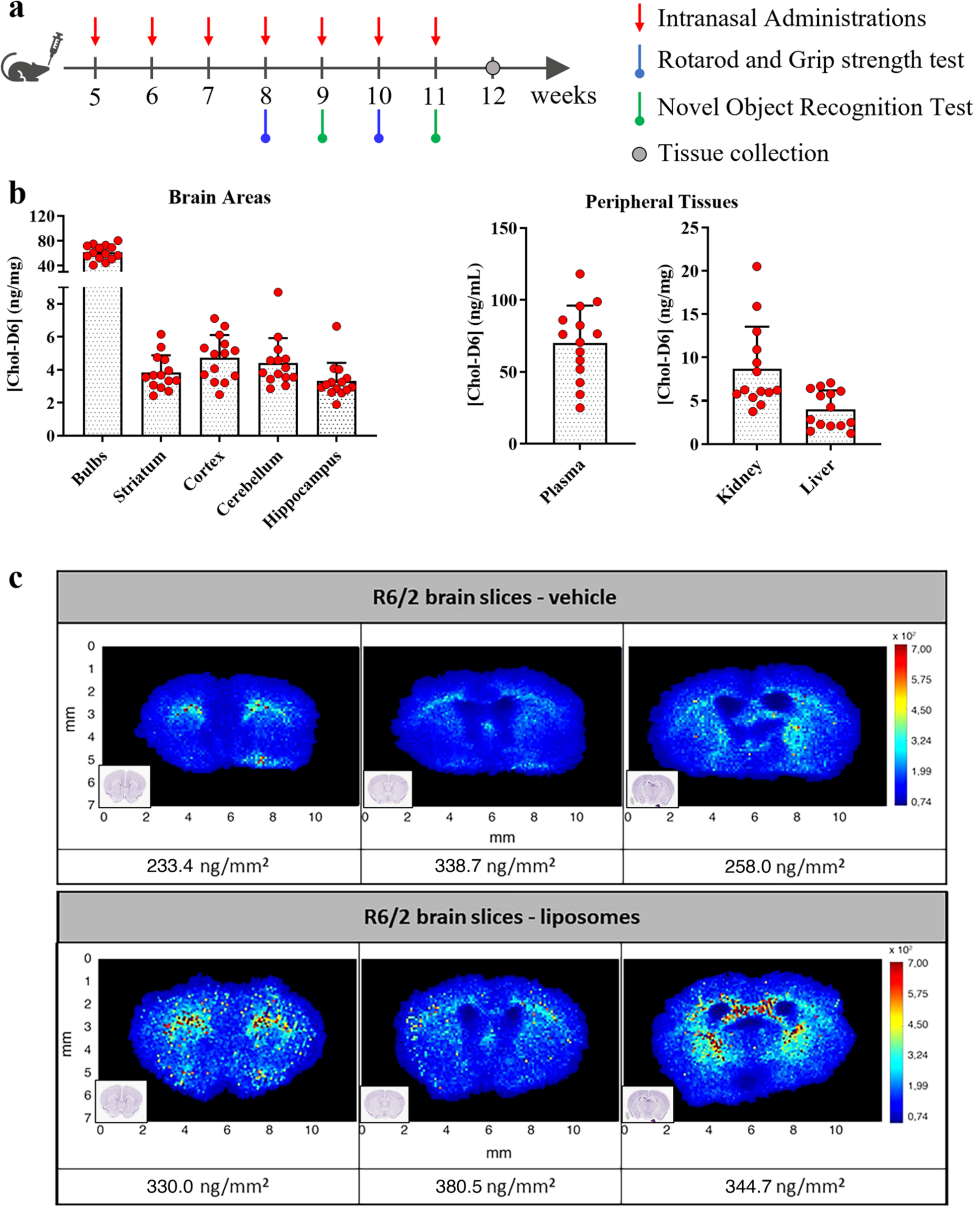
Mass-spectrometry analysis of chol-D6 in WT and R6/2 mice following sub-chronic intranasal treatment. **a** Experimental paradigm used in the sub-chronic studies. R6/2 mice were treated with chol-D6-enriched liposomes from 5 to 11 weeks of age with one intranasal administration each week (200 µg chol-D6/IN). PBS was used as a control. Behavioral tests were conducted at different time points. At 12 weeks, brains, peripheral tissues, and plasma were collected. **b** Levels of chol-D6 in bulbs, striatum, cortex, cerebellum, hippocampus, plasma, liver, and kidney of R6/2-liposomes mice measured by LC–MS analysis (*n* = 14/tissue). Each dot corresponds to an animal. The levels of chol-D6 in tissues from both WT-vehicle and R6/2-vehicle groups were not detectable. Results are shown as mean ± standard deviation. **c** Upper panel: representative IMS results of brain slices from an R6/2-vehicle mouse (three brain regions from the same animal); lower panel: representative IMS results of brain slices from an R6/2-liposomes mouse (three brain regions from the same animal). Cholesterol concentrations are expressed in ng/mm^2^ and reported below each image. The IMS analysis provides a proof-of-concept visualization of the spatial distribution of total cholesterol (endogenous + exogenous) within brain sections of treated animals

To evaluate the accumulation profile of chol-D6 after repeated administrations and to determine whether exogenous cholesterol accumulation in the brain could ameliorate the HD phenotype in the R6/2 mouse model, we performed a sub-chronic treatment study in R6/2 mice. One group of R6/2 mice received chol-D6-enriched liposomes (200 μg per intranasal administration) (R6/2-liposomes), while a second group of R6/2 mice and a group of WT mice received PBS (R6/2- or WT-vehicle). Each animal received seven intranasal administrations, one per week, from 5 to 11 weeks of age. The WT and R6/2 animals were subjected to various behavioral tests to assess cognitive and motor deficits, as well as muscular strength. At the end of the sub-chronic treatment (12 weeks of age), all animals were sacrificed, and tissues were collected (Fig. 3a).

As expected, chol-D6 showed a predominant accumulation in the olfactory bulbs in mice of the R6/2-liposomes group, reflecting their proximity to the administration site. All analyzed brain regions (bulbs, striatum, cortex, cerebellum and hippocampus) exhibited similar accumulation patterns, demonstrating that exogenous cholesterol can reach and accumulate throughout the brain after repeated intranasal administrations (Fig. 3b). Compared with the single intranasal treatment data, these results indicate that the delivered cholesterol can accumulate in brain areas following seven intranasal administrations, with a slow elimination rate. In contrast, chol-D6 levels in plasma and peripheral tissues, such as the kidney and liver, remained consistent with those observed after a single intranasal administration. This indicates that exogenous cholesterol is continuously cleared from peripheral tissues without significant accumulation (Fig. 3b).

We developed an IMS-based method to map cholesterol distribution in brain sections, complementing the LC–MS approach. Given that chol-D6 is required as the internal standard for imaging, we chose to administer non-tagged cholesterol and assess its distribution in the brain after sub-chronic treatment. As a proof of concept, we quantified total cholesterol levels in brain slices from R6/2 animals treated with liposomes enriched with non-tagged cholesterol, using IMS, and compared them to levels in the R6/2-vehicle animals (Fig. 3c). To obtain semi-quantitative information about cholesterol levels in the brain, we generated a standard curve by spotting increasing concentrations of cholesterol directly onto the AP-MALDI (atmospheric pressure matrix-assisted laser desorption/ionization) target, using chol-D6 as an internal standard, thus enabling a semiquantitative analysis in IMS (Fig. S2a, b; details of the quantitative approach are listed in Table S2). The proof-of-concept IMS results showed that R6/2-liposomes exhibited higher total cholesterol levels compared to untreated R6/2-vehicle (Fig. 3c). These promising results pave the way for future studies that focus on analysing the spatial distribution of cholesterol and its metabolites in brain sections using IMS as a complementary method alongside LC–MS analysis.

### Cholesterol administration improves behavioral performance of R6/2 mice

**Fig. 4:**
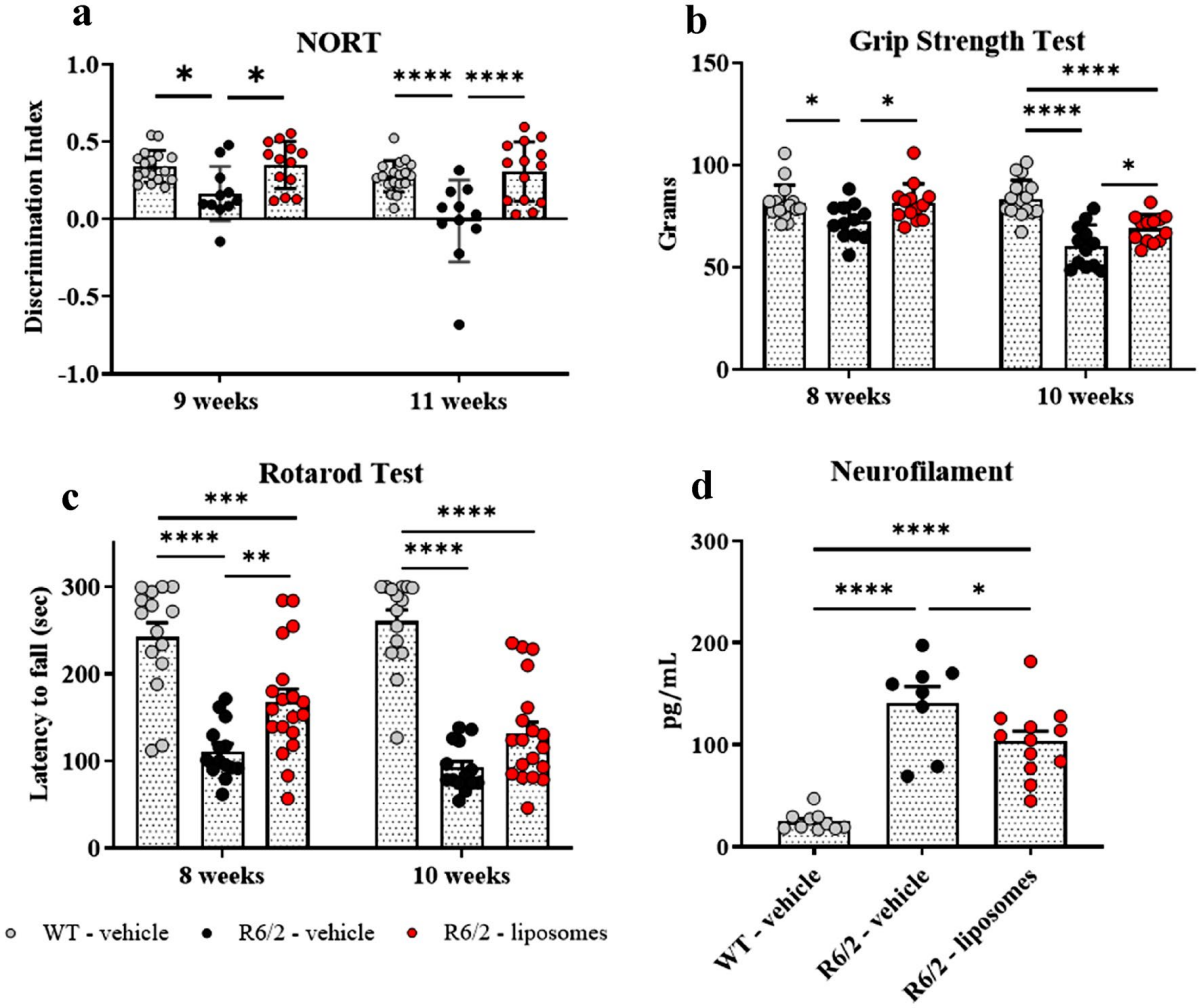
Behavioral performance and neurofilament analysis in WT and R6/2 mice following sub-chronic intranasal treatment. **a** The NORT was performed at 9 and 11 weeks of age (*n* = 17–18 mice for WT-vehicle; 11 for R6/2-vehicle; and 13–14 for R6/2-liposomes). **b** At 8 and 10 weeks, the grip strength test was performed (*n* = 16 mice for WT-vehicle; 12 for R6/2-vehicle; 13 for R6/2-liposomes). **c** Results of the rotarod test performed at 8 and 10 weeks of age (*n* = 15 mice for WT-vehicle; 14 for R6/2-vehicle; and 19 for R6/2-liposomes). **d** Plasma neurofilament concentrations after sub-chronic treatment (*n* = 10 mice for WT-vehicle; 8 for R6/2-vehicle; 12 for R6/2-liposomes). Each dot represents an animal. Results are shown as mean ± standard deviation. Statistics: mixed-effects analysis test with post-hoc Tukey (*P < 0.05; **P < 0.01; ***P < 0.001; ****P < 0.0001) or one-way ANOVA test with post-hoc Tukey test (*P < 0.05; ****P < 0.0001)

The effects of cholesterol supplementation on memory and cognitive functions were assessed by the NORT at 9 and 11 weeks of age, after sub-chronic intranasal administration of cholesterol-enriched liposomes. As expected, the R6/2-vehicle failed to distinguish the novel object, exhibiting memory impairment at both time points (Fig. 4a). In contrast, the R6/2 mice treated intranasally with cholesterol-enriched liposomes showed no memory loss and performed comparably to the WT-vehicle group, demonstrating a striking reversal of cognitive decline (Fig. 4a).

To assess muscular strength and motor coordination, grip strength and rotarod tests were conducted at 8 and 10 weeks of age (Fig. 4b, c). At 8 weeks, the WT-vehicle and the R6/2-liposomes groups showed comparable results, with significant increases in muscular strength compared to the R6/2-vehicle. By 10 weeks, the R6/2 mice treated with liposomes continued to show improved muscular strength compared to the R6/2-vehicle group, though they did not reach the performance level of WT-vehicle animals (Fig. 4b). The grip strength test results indicated that cholesterol supplementation can counteract early-stage symptoms of the disease. Additionally, data from the rotarod test revealed that exogenous cholesterol supplementation slowed the progressive motor impairment characteristic of the R6/2 mouse strain. At 8 weeks of age, the R6/2-vehicle animals exhibited a significant motor deficit as the disease progressed, performing markedly worse than the WT-vehicle animals (Fig. 4c). Cholesterol-enriched liposome brain supply induced a significant improvement in motor abilities of R6/2 mice compared to the R6/2-vehicle animals (Fig. 4c). However, as the disease progressed to 10 weeks, both R6/2-liposomes and R6/2-vehicle groups exhibited a significant decline in performance compared to the WT-vehicle animals, indicating that the beneficial effects of the treatment diminished with advancing of disease severity (Fig. 4c). No effects on body weight were observed (Fig. S3).

### Cholesterol supplementation decreases plasma NfL level

NfL has been recognized as a biomarker for neurodegeneration in several neurological disorders, including HD [27–31], where it serves as an indicator of axonal damage. NfL levels have been used as an endpoint in numerous clinical trials for CNS diseases [32]. To assess the extent of neurodegeneration following sub-chronic treatment with cholesterol-enriched liposomes, we measured NfL levels in plasma samples collected at the time of sacrifice at 12 weeks of age. The NfL concentration in the R6/2-liposomes group was significantly lower (by 27%) than that in the R6/2-vehicle group (Fig. 4d). These findings suggest that cholesterol supplementation to the brain reduces NfL production, indicating its efficacy in slowing and/or reducing axonal damage in HD mice.

### Cholesterol supplementation reduces muHTT aggregates in the R6/2 brain

**Fig. 5:**
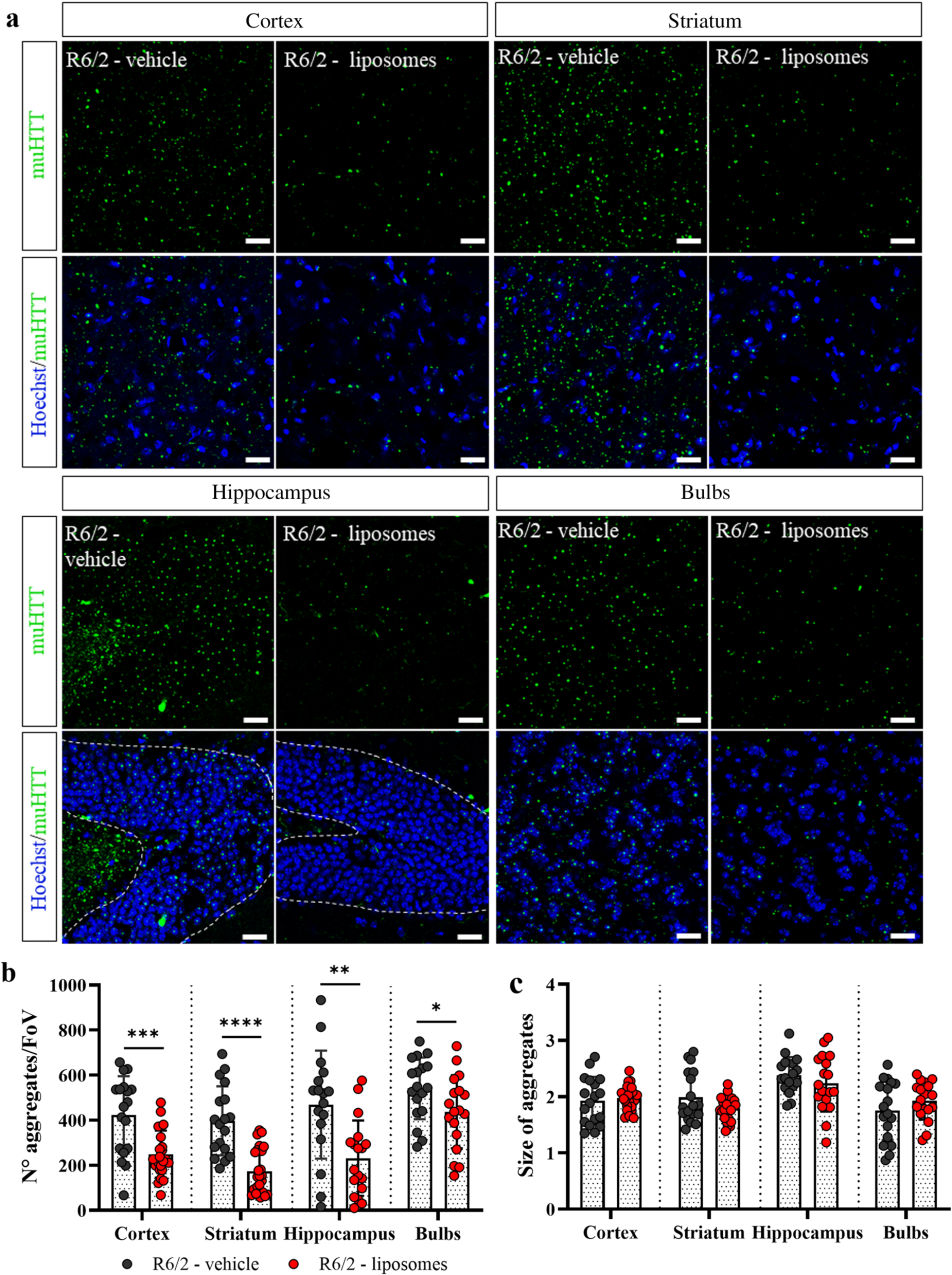
Immunostaining analysis of mutant huntingtin (muHTT) aggregates**. a** Immunostaining of muHTT (EM48 antibody, green) in the cortex, striatum, hippocampus, and bulbs of R6/2-liposomes mice and R6/2-vehicle mice after sub-chronic treatment. Hoechst (blue) was used to counterstain nuclei. **b**, **c** Quantification of the number and size of muHTT aggregates. Scale bars, 20 μm. *n* = 3 mice for R6/2-vehicle, 4 mice for R6/2-liposomes. Data are shown as mean ± standard deviation. Each dot represents an image. Student’s *t*-test; *P < 0.05, **P < 0.01, ***P < 0.001; ****P < 0.0001

The presence of intracellular aggregates of muHTT is a key neuropathological feature of HD [33]. To evaluate whether sub-chronic intranasal delivery of cholesterol affects muHTT aggregation, we performed immunofluorescence staining with the EM48 antibody, which specifically recognizes the aggregation-prone polyQ tract, at 12 weeks of age (Fig. 5a). The immunofluorescence staining showed that the number of aggregates was significantly reduced in R6/2 mice treated with liposomes compared to the R6/2-vehicle group, in all brain regions analyzed, including striatum, cortex, hippocampus, and olfactory bulbs (Fig. 5a, b). However, the treatment did not affect the size of the muHTT aggregates (Fig. 5c).

### Analysis of cholesterol precursors and metabolite in the striatum

To determine whether cholesterol supplementation can influence endogenous cholesterol metabolism, we analyzed the levels of cholesterol precursors desmosterol and 7-dehydrocholesterol, conventionally involved in the Bloch and Kendutsch-Russel pathways, respectively [6], and the level of the neuronal-specific cholesterol catabolite 24S-hydroxycholesterol (24S-OHC), in the striatum of WT and R6/2 animals undergoing sub-chronic treatment.

The R6/2-vehicle mice exhibited a significantly lower desmosterol concentration than the WT-vehicle and the R6/2-liposomes mice (Fig. S4a). No significant differences in 7-dehydrocholesterol were found among the three groups of animals (Fig. S4b). Gene expression analysis did not reveal significant changes in the expression of genes related to cholesterol synthesis (*Srebp2 Hmgcr, Fdft1, Mvk* and *Dhcr7*), efflux (*Abca1* and *ApoE*), and metabolism (*Cyp46a1* and *Cyp11a1*) in the striatum between R6/2 animals undergoing sub-chronic intranasal cholesterol treatment and PBS treatment (Fig. S5).

## Discussion

The intricate relationship between brain function and intracerebral production of cholesterol has been extensively studied in health and disease. In HD, reduced cholesterol biosynthesis has been systematically measured and evaluated in various animal models over the past 25 years [2–6]. Restoring cerebral cholesterol homeostasis has been demonstrated to significantly improve the main symptoms of the disease in several models, using different approaches (i.p. injection of cholesterol-loaded nanoparticles or striatal infusion of cholesterol via minipumps, or AAV-SREBP2 [sterol regulatory element binding protein 2]) [6–8, 10–13]. While these approaches are promising, they have limitations due to their invasiveness. To address this issue, we recently developed a method to increase cerebral cholesterol levels through intranasal administration of liposomes composed solely of cholesterol and phosphatidylcholine [9].

In this study, we investigated whether intranasal administration of cholesterol-enriched liposomes could counteract symptoms in the rapidly progressing R6/2 mouse model. We first analyzed the pharmacokinetic profile of exogenous cholesterol (chol-D6) following a single intranasal dose of cholesterol-enriched liposomes. The pharmacokinetic analysis showed that chol-D6 achieved steady-state concentrations in various brain regions for up to 10 days post-administration. Notably, the pharmacokinetic profiles were similar between WT and R6/2 mice, indicating that the disease state does not significantly impact the distribution and retention of exogenous cholesterol in the brain. Moreover, IMS analysis showed that exogenous cholesterol was distributed in cortical and basal ganglia regions following intranasal treatment. Chronic treatment, involving weekly intranasal administration from 5 to 11 weeks of age, resulted in a significant accumulation of chol-D6 throughout the brain in R6/2 mice. This accumulation occurred without excessive build-up in peripheral tissues, highlighting the targeted nature of this delivery method.

An important aspect of our findings is that a relatively small increase in brain cholesterol-D6 (~ 4–5 ng/mg), corresponding to only 0.025% of total endogenous brain cholesterol (~ 10–20 µg/mg), leads to substantial improvements in behavioural outcomes and neuropathological markers. This apparent discrepancy can be explained by the compartmentalisation of brain cholesterol into functionally distinct pools. Approximately 70% of brain cholesterol resides in metabolically inert myelin membranes with slow turnover (half-life of ~ 5 years in humans), whereas the remaining ~ 30% constitutes a metabolically active pool in neuronal and glial plasma membranes that is essential for synaptic function [34, 35]. This active pool supports lipid raft formation, enabling the organization and clustering of neurotransmitter and neurotrophin receptors, ion channels, and signalling molecules critical for synaptic transmission [36, 37]. Our findings indicate that restoring cholesterol to this small but metabolically active pool is sufficient to ameliorate synaptic dysfunction and its downstream consequences, rather than affecting the bulk cholesterol content. This interpretation is consistent with the observation that the adult mouse brain synthesizes only ~ 35 µg cholesterol/day, corresponding to ~ 70 ng/mg per day [38] with a fractional daily turnover of only ~ 0.5% of the total cholesterol pool (corresponding to ~ 15 µg/mg), indicating that even modest supplementation can impact the active cholesterol pool.

Additionally, the study confirmed the safety of this approach, as no adverse effects on animal welfare were observed in WT mice undergoing sub-chronic treatment. These findings reinforce the potential for intranasal cholesterol administration to advance to clinical practice [39], specifically for accelerating Phase 1 clinical trials. The comparable pharmacokinetics and cholesterol accumulation in brain regions between healthy and transgenic mice, coupled with the absence of peripheral accumulation, suggest favourable safety and tolerability profiles for chronic cholesterol administration. Notably, in all cholesterol-raising trials mentioned earlier (i.p. injection of cholesterol-loaded nanoparticles or striatal infusion of cholesterol via minipumps, or AAV-SREBP2), regardless of the treatment, animal model, or timing of delivery, the administration of exogenous cholesterol or improvement of its biosynthesis in the HD mouse brain completely normalized cognition [8, 10–13]. In the present study, the accumulation of chol-D6 in the striatum, hippocampus and cortex was found to be associated with a complete recovery of cognitive functions. This finding further demonstrated that safer delivery of cholesterol via intranasal administration can effectively counteract cognitive deficits in HD, reinforcing the hypothesis that cholesterol has a potential neuroprotective and neurorestorative role in modulating synaptic plasticity and maintaining neuronal integrity in these vulnerable areas [40, 41].

In the three approaches mentioned above [7–11], only the high cholesterol dose administered using osmotic minipumps was able to improve coordination and motor ability in R6/2 mice. In our study, although we did not achieve the same high dose of cholesterol, the R6/2 mice treated with liposomes also showed improved coordination and motor ability compared to the R6/2-vehicle group. This suggests that intranasal cholesterol administration causes immediate bioavailability of cholesterol and can be used to restore brain function. In addition, the grip strength test results indicated that cholesterol supplementation can counteract early-stage strength symptoms of the disease. Together, these results suggest that intranasal cholesterol administration can improve multiple aspects of the HD phenotype.

Following intranasal administration of cholesterol, we also analyzed the cholesterol precursors using mass spectrometry. Our results confirmed that a dose of exogenous cholesterol supplementation in the brain is required to stimulate the endogenous synthesis of cholesterol via the desmosterol precursor pathway [42]. However, this supplementation did not enhance the transcription of genes coding the enzymes involved in cholesterol homeostasis. 24S-OHC is the predominant metabolite of brain cholesterol and can be measured in the blood and cerebrospinal fluid. In humans, but not in rodents, 99% of the 24S-OHC found in plasma is of the cerebral origin [43]. There is a consensus that 24S-OHC levels are reduced in some neurodegenerative disorders. However, results of 24S-OHC levels in the plasma of individuals with such disorders have often been inconsistent in cross-sectional studies [44, 45]. This inconsistency might be due to variations in study design, patient populations, or differences in the method used for measuring 24S-OHC. In our study we found a reduction of 24S-OHC concentration in the striatum of R6/2-liposomes and R6/2-vehicle groups compared to the WT-vehicle group (Fig. S4c), consistent with our earlier findings of reduced 24S-OHC in the brains of HD mice [10, 11, 13, 26]. Other laboratories have found increases in HD mice or no significant differences between WT and HD mice [15, 46]. Variations in tissue preparation methods (e.g., blood perfusion) and measurement techniques can influence these results. A longitudinal study is currently ongoing to evaluate the prognostic value of plasma levels of 24S-OHC as an early biomarker in HD (NCT04257513).

Previous studies showed that all cholesterol-based strategies used so far can promote the clearance of muHTT aggregates in the HD mouse brain [10–13]. Consistently, our results showed that intranasal cholesterol administration effectively reduced muHTT aggregates in the olfactory bulbs, striatum, hippocampus, and cortex, though their sizes were not affected. These findings demonstrate that the intranasal administration of cholesterol-enriched liposomes is sufficient to promote the clearance of muHTT in these key brain regions [6, 10–13]. A recent study in vitro further supported the relationship between muHTT aggregates and cholesterol by showing that increasing cholesterol content in a surrogate membrane decreased muHTT fibril formation and reduced the interaction of muHTT with the membranes [47]. Therefore, cholesterol supplementation to the brain appears to inhibit muHTT aggregate formation at multiple levels.

Elevated NfL concentrations have been detected in plasma and CSF of HD patients before symptom onset, serving as a predictor of clinical status, disease progression, and brain atrophy [48–50]. Morever, NfL levels have been used as endpoints in numerous clinical trials for CNS diseases [32]. Our results showed that cholesterol supplementation significantly reduced NfL production, suggesting that exogenous cholesterol may help mitigate axonal damage and slow neurodegeneration in HD mice. This is the first time a link is made between cholesterol administration and the reduction of a pharmacodynamic biomarker used to monitor disease progression. This finding is relevant in future clinical trials to evaluate the safety and efficacy of cholesterol-based strategies in HD patients. Indeed, in clinical trials for various neurodegenerative diseases, including multiple sclerosis, amyotrophic lateral sclerosis and HD, plasma NfL levels have been used as an endpoint to assess whether to proceed with further trials. Recently, the FDA approved the antisense oligonucleotide Tofersen for treating amyotrophic lateral sclerosis based on its ability to reduce plasma NfL levels [32]. The reduction of NfL registered in our study is particularly significant given its emerging role as both a prognostic marker and a potential endpoint in clinical trials for neurodegenerative diseases.

## Conclusions

In conclusion, we demonstrate the full efficacy of the intranasal route in delivering cholesterol to the HD brain and ameliorating multiple HD hallmarks. This method offers several advantages as it is non-invasive, bypasses the BBB, and holds potential for patient self-administration. Additionally, the liposome formulation is biocompatible, biodegradable, easily scalable, and optimized for Good Manufacturing Process production, underscoring its safety profile and potential for clinical translation. The new observations presented here reinforce the potential for intranasal cholesterol administration to advance to clinical practice [39], providing a strong rationale for accelerating Phase 1 clinical trials. The comparable pharmacokinetics and cholesterol accumulation in brain regions observed between healthy and transgenic mice, along with the absence of peripheral accumulation, suggest a favorable safety and tolerability profile for chronic cholesterol administration. These findings position intranasal cholesterol delivery as a promising therapeutic approach not only for HD but also for other neurodegenerative disorders associated with brain cholesterol deficits, including Alzheimer’s disease and Parkinson’s disease [51, 52]. This study lays the groundwork for future investigations to optimize this delivery method, explore its efficacy in additional HD animal models, and eventually evaluate its potential in human patients.

## Supplementary Information

Additional file 1. **Fig. S1**. Effects of sub-chronic intranasal cholesterol-enriched liposomes on Cholesterol-D6 levels, body weight, and behavior in WT mice. **Fig. S2**. Mass spectrometry spectra and calibration curve for cholesterol quantification via the imaging approach. **Fig. S3**. Body weight monitoring of R6/2 and WT mice during sub-chronic intranasal treatments. **Fig. S4**. Desmosterol, 7-dehydrocholesterol and 24S-hydroxycholesterol profiles after sub-chronic treatment in R6/2 and WT mice. **Fig. S5**. Cholesterol precursors and metabolite profile in the striatum. **Table S1**. MRM transitions for cholesterol-D6 and β-sitosterol quantitation and relative collision energies (CE). **Table S2**. MRM transitions for cholesterol metabolites quantitation and relative collision energies (CE). **Table S3**. List and sequence of primers used in this study for gene expression analysis. **Table S4**. Number of animals used in each read-out performed in this study's trials.

## Data Availability

This study does not include data deposited in public repositories. Data are available from the corresponding author upon reasonable request.

## Abbreviations

HD: Huntington’s disease
BBB: Blood-brain barrier
muHTT: Mutant Huntingtin
NORT: Novel object recognition test
IMS: Imaging mass spectrometry
LC-MS: Liquid chromatography-mass spectrometry
NfL: Neurofilament light
DCM: Dichloromethane
AGC: Automatic gain control
