# Supplementary material for: Cholesterol nose-to-brain delivery as a possible therapeutic strategy in Huntington’s disease
Source: Transl Neurodegener. 2026 Aug 4;15:37. doi: 10.1186/s40035-026-00569-x (PMC13435458; doi:10.1186/s40035-026-00569-x)
Supplement: Supplementary file 1 [file 40035_2026_569_MOESM1_ESM.pdf]

SUPPLEMENTARY MATERIAL

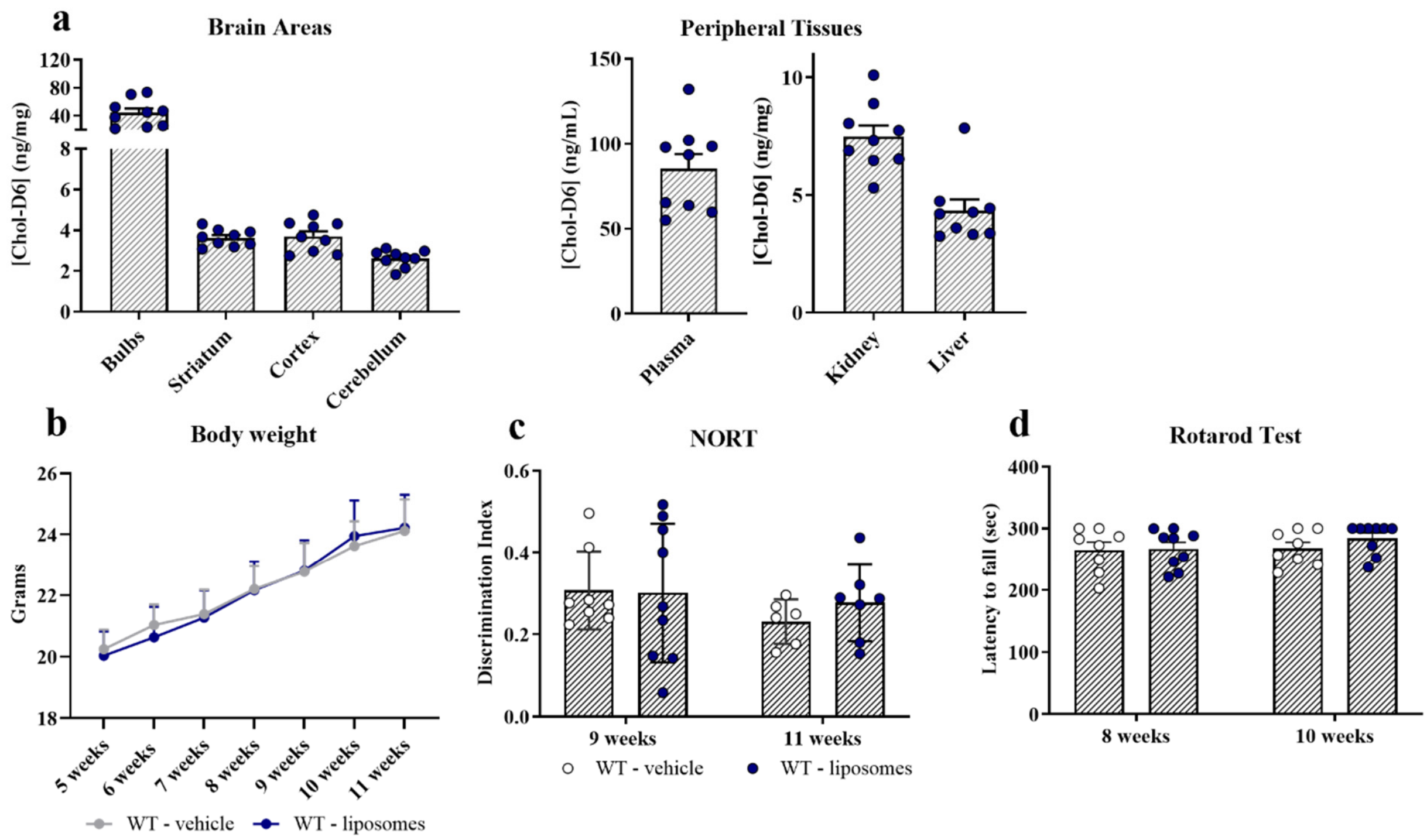

Fig. S1. Effects of sub-chronic intranasal cholesterol-enriched liposomes on Cholesterol-D6 levels, body weight, and behavior in WT mice. **(a)** Levels of chol-D6 in brain areas (olfactory bulbs, striatum, cortex, and cerebellum) and peripheral tissues (plasma, kidney, and liver) in WT mice treated with chol-D6-based liposomes from 5 to 11 weeks of age with one IN/week (200 µg chol-D6/IN) (*n*= 9). (**b**) Animal weight trend during disease progression from the fifth to the twelfth weeks of age. Grey line: WT-vehicle, blue line: WT-liposomes. (**c**) NORT was performed at 9 and 11 weeks of age. (**d**) Rotarod test was performed at 8 and 10 weeks of age. Each dot **(a, c-d)** corresponds to the value obtained from an animal. White dots: WT-vehicle; blue dots: WT-liposomes. **(a-d)** Data are from one trial as graphs with mean ± standard deviation (M±SD). WT-vehicle = 8, WT-liposomes = 9. Statistics: two-way ANOVA with Tukey post-hoc test.

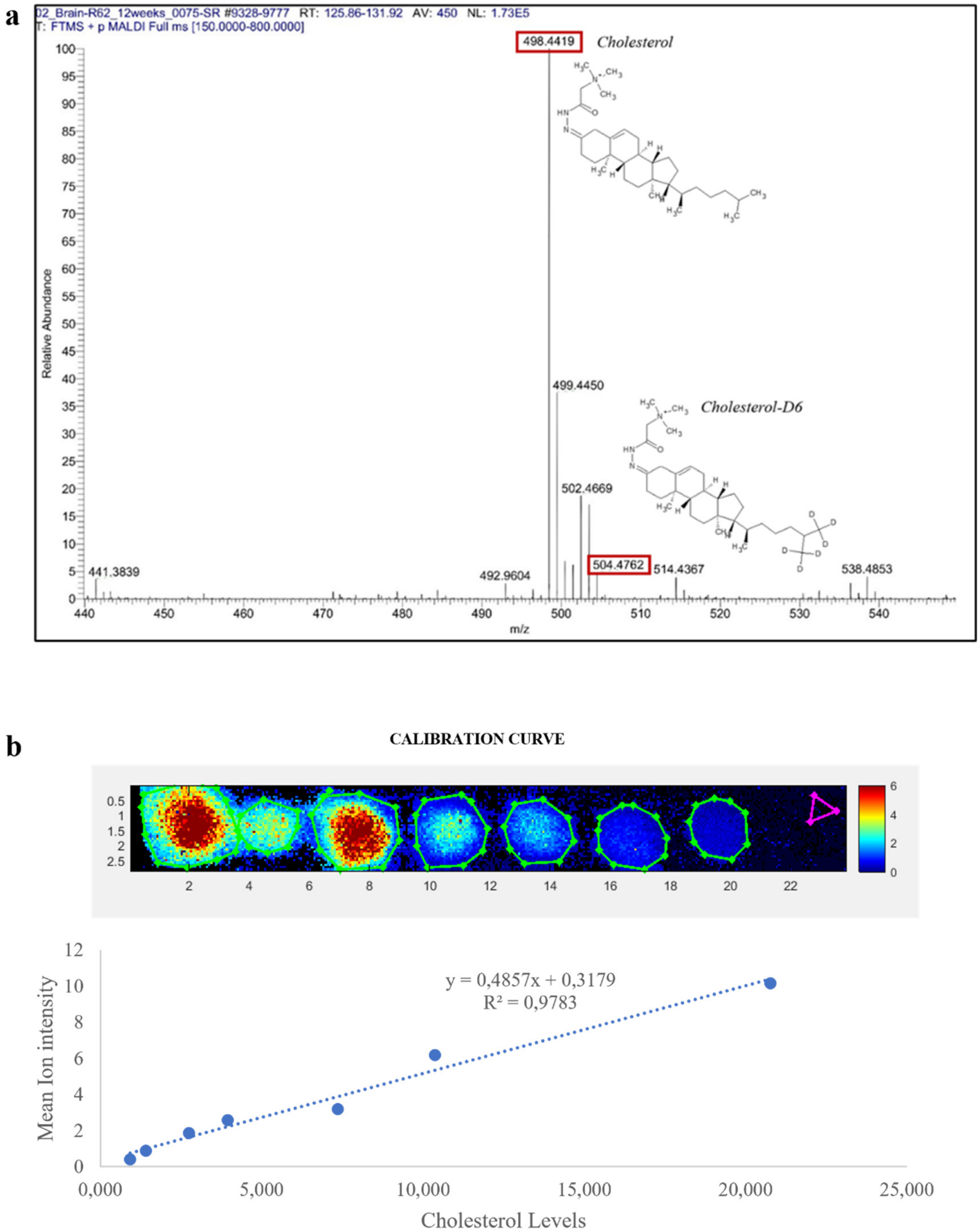

Fig. S2. Mass spectrometry spectra and calibration curve for cholesterol quantification via the imaging approach. **(a)** Mean spectrum (450 scans) of WT brain slice obtained during an IMS experiment, highlighting cholesterol signal (m/z 498.4419) and chol-D6 signal (m/z 504.4762) after derivatization using Girard's T reagent. **(b)** The calibration curve for the IMS experiment was created by plotting the cholesterol signal intensity from manually selected regions of interest (ROIs) of each spotted calibrator against the signal of chol-D6 sprayed as an internal standard. The concentration is expressed as ng/mm² from 50 to 1000, and the ROIs of each brain slice were selected manually. The

concentration is derived by plotting the ratio of cholesterol signal to IS for each brain slice against the calibration curve.

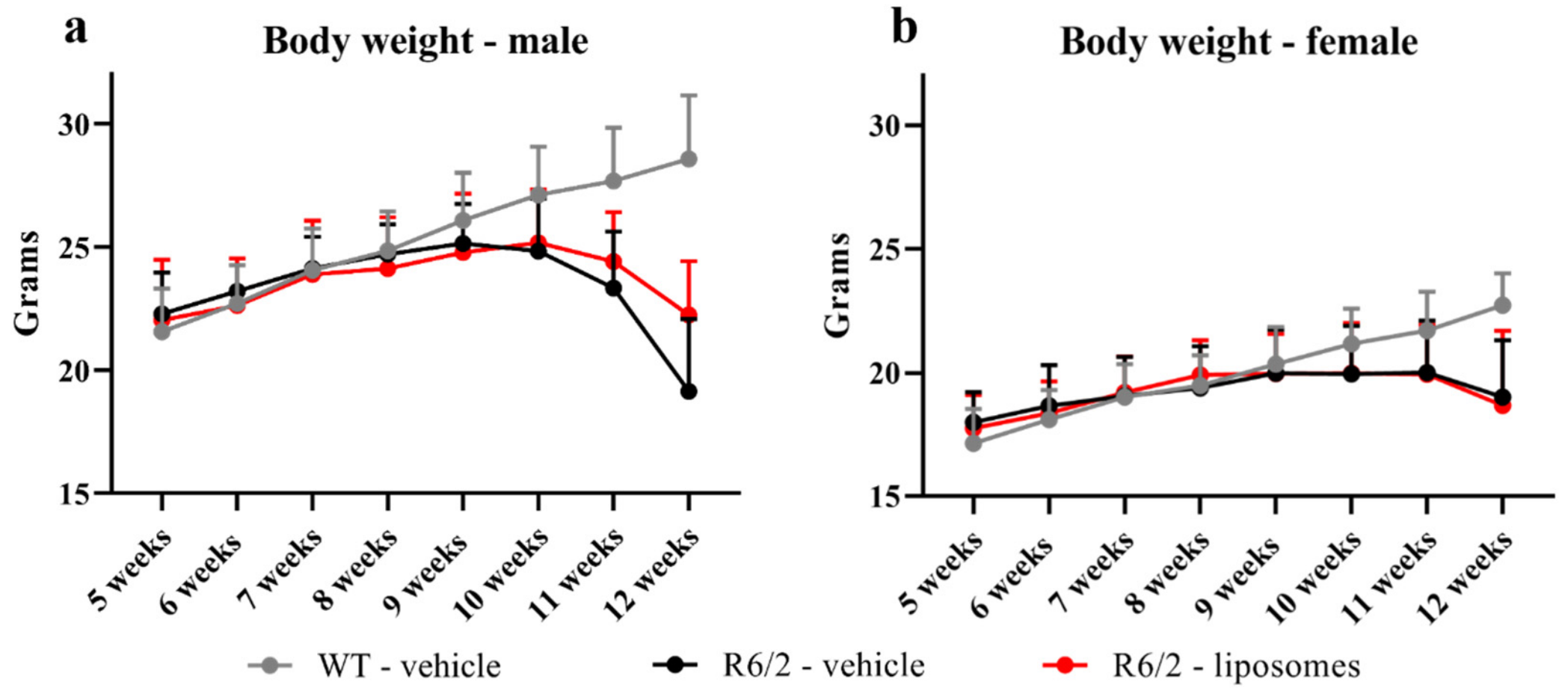

Fig. S3. Body weight monitoring of R6/2 and WT mice during sub-chronic intranasal treatments. **(a-b)** The weight trend of animals during disease progression, from the fifth to the twelfth weeks of age, in **(a)** males and **(b)** females. Grey line: WT-vehicle, black line: R6/2-vehicle, red line: R6/2-liposome. Data are from five independent trials as graphs with mean ± standard deviation (M±SD). Statistics: two-way ANOVA with Tukey post-hoc tests (male: at tenth week, WT-vehicle vs. R6/2-vehicle and -liposomes, $P<0.01$; at eleventh week, WT-vehicle vs. R6/2-vehicle and -liposomes, $P<0.0001$; at twelfth week, WT-vehicle vs. R6/2-vehicle and -liposomes, $P<0.0001$; R6/2-vehicle vs R6/2-liposomes, $P<0.01$. Female: at tenth week, WT-vehicle vs. R6/2-vehicle and -liposomes, $P<0.05$; at eleventh week, WT-vehicle vs. R6/2-vehicle and -liposomes, $P<0.01$; at twelfth week, WT-vehicle vs. R6/2-vehicle and -liposomes, $P<0.0001$).

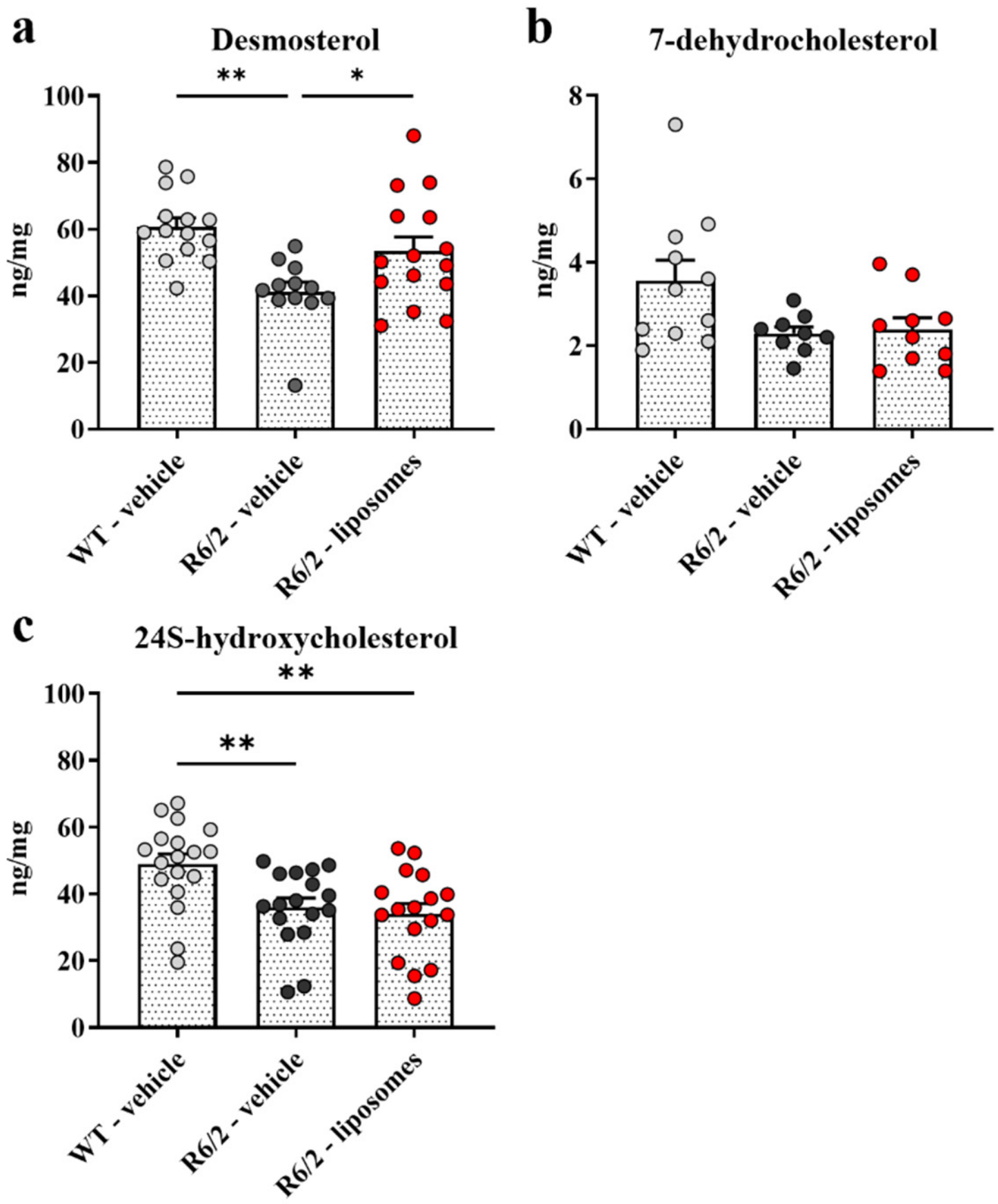

Fig. S4. Desmosterol, 7-dehydrocholesterol and 24S-hydroxycholesterol profiles after sub-chronic treatment in R6/2 and WT mice. **(a-c)** Concentrations of **(a)** desmosterol, **(b)** 7-dehydrocholesterol, and **(c)** 24S-hydroxycholesterol in striatum areas after sub-chronic treatment (WT-vehicle = 14; R6/2-vehicle = 12; R6/2-liposomes = 15 for desmosterol; WT-vehicle = 11; R6/2-vehicle = 9; R6/2-liposomes = 10 for 7-dehydrocholesterol; WT-vehicle = 18; R6/2-vehicle = 17; R6/2-liposomes = 17 for 24S-hydroxycholesterol). Each dot corresponds to the value obtained from each animal. White dots: WT-vehicle, black dots: R6/2-vehicle, red dots: R6/2-liposomes. Data are graphed with mean ± standard deviation (M±SD). Statistics: one-way ANOVA test with post-hoc Tukey test (*P<0.05; **P<0.01).

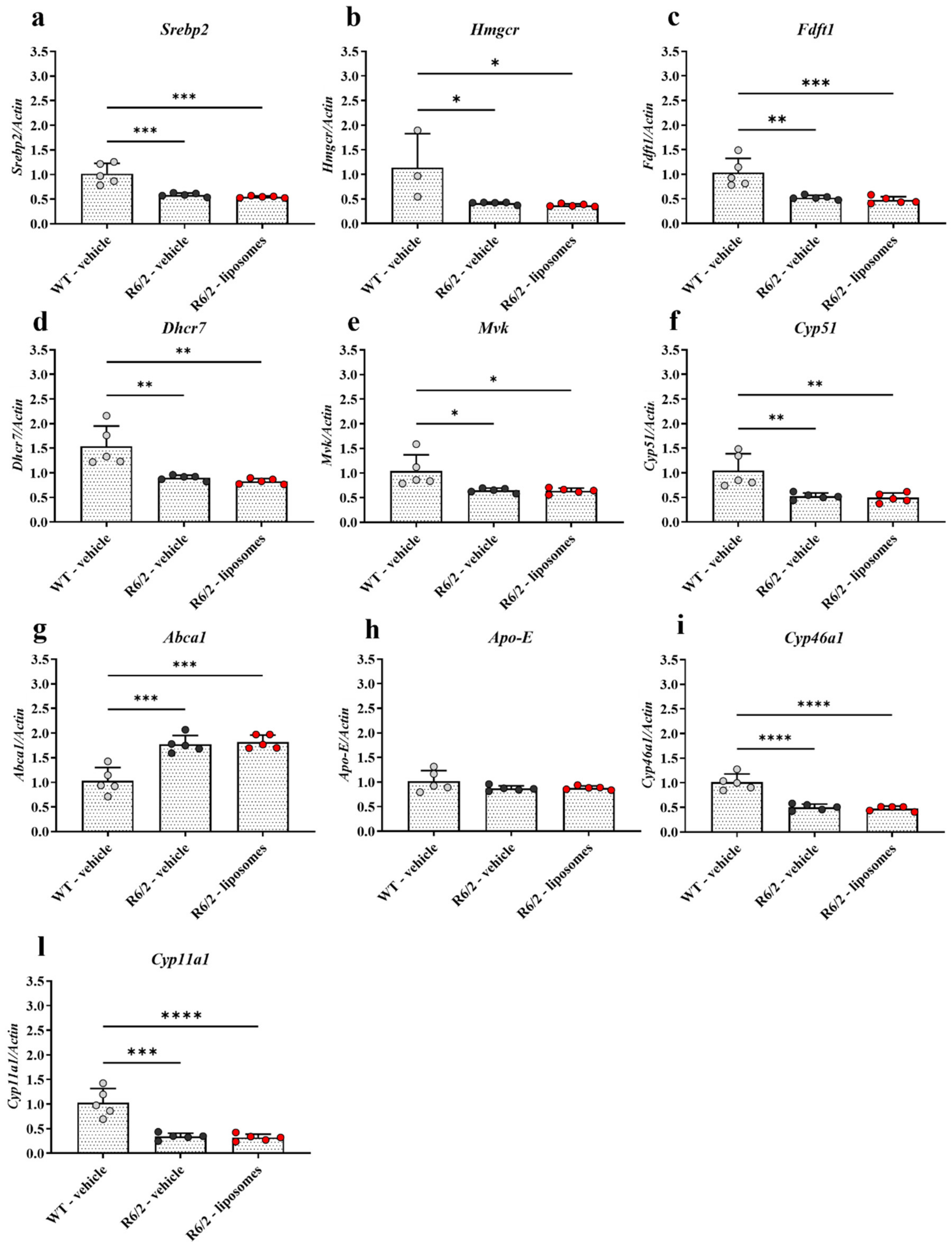

Fig. S5. Cholesterol precursors and metabolite profile in the striatum**. (a)** mRNA levels of the transcription factor Srebp2. (**b-f)** mRNA levels of several enzymes involved in cholesterol synthesis. (**g-h)** mRNA levels of enzymes involved in cholesterol efflux. (**i-l)** mRNA levels of enzymes involved in cholesterol catabolism (*n* = 3-5

animals/gene/group). Each dot corresponds to the value obtained from an animal. White dots: WT-vehicle, black dots: R6/2-vehicle, red dots: R6/2-liposomes. Data are graphed with mean ± standard deviation (M±SD). Statistics: one-way ANOVA test with post-hoc Tukey test (**P*<0.05; ***P*<0.01; ****P*<0.001; *****P*<0.0001).

Table S1. MRM transitions for cholesterol-D6 quantitation and relative collision energies (CE).

Analyte	Quantifier	CE	Qualifier	CE
Cholesterol-D6	375.3 → 152.1	30	375.3 → 167.1	30
B-sitosterol	397.3 → 147.1	30	397.3 →161.1	30

Table S2. MRM transitions for cholesterol metabolites quantitation and relative collision energies (CE).

Analyte	Quantifier	CE	Qualifier	CE
7α-dihydroxycholesterol [7αHC]	383.5 → 159.3	27	383.5 → 105.1	45
24S-hydroxycholesterol [24S-OHC]	367.3 → 159.1	28	363.3 → 133.1	25
24-hydroxycholesterol-D_7 [24-OHC-D_7]	374.1 → 159.1	23	374.1 → 147.1	25
Lathosterol-D_7	376.3 → 161.1	24	376.3 → 105.1	45
25-hydroxycholesterol [25-OHC]	367.1 → 147.1	25	367.1 → 159.2	25
25-hydroxycholesterol-D_6 [25-OHC-D_6]	373.3 → 147.1	24	373.3 → 161.1	23
27-hydroxycholesterol [27-OHC]	385.2 → 161.1	21	385.2 → 135.1	24
Desmosterol [Desm]	367.2 → 147.1	24	367.2 → 95.1	30
Desmosterol-D_6 [Desm-D_6]	373.2 → 147.1	24	373.2 → 161.1	23
Lanosterol [Lan]	409.2 → 95.1	35	409.2 → 109.2	25
22-hydroxycholesterol [22-OHC]	367.3 → 159.3	25	367.3 → 91.2	54
7-dehydrocholesterol [7-dehydrochol]	367.2 → 159.1	21	367.2 → 95.3	34

Table S3. List and sequence of primers used in this study for gene expression analysis.

**Primer**	**Sequence**
mActin-FW	AGTGTGACGTTGACATCCGTA
mActin-RV	GCCAGAGCAGTAATCTCCTTCT
mFDFT1-FW	ACTCAGCAGCAGCTTGAAGACC
mFDFT1-RV	TGTCATCCTCCACTGTATCCAG
mHMGCR-FW	GGAGCATAGGCGGCTACA
mHMGCR-RV	ACCACCCACGGTTCCTATCT
SREBP2-FW	GCCTCTCCTTTAACCCCTTG
SREBP2-RV	CCAGTCAAACCAGCCCCCAG
Dhcr7-FW	CAAGACACCACCTGTGACAGCT
Dhcr7-RV	CTGCTGGAGTAATGGCACCTTC
MVK-FW	CCCTCCTGAAGCCAGGTCTA
MVK-RV	TGGTCTCCCAGCAGTCAAAC
CYP51-FW	GGCAGAGCGCTTGGACTTTA
CYP51-RV	ATGACGCCCAGCTCCAAATG
ABCA1-FW	GTCAGCTGTTACTGGAAGTGG
ABCA1-RV	CGCCGGGAGTTGGATAACGG
mAPOE-FW	GACAGATCAGCTCGAGTGGC
mAPOE-RV	CTTCCGTCATAGTGTCCTCCAT
mCYP46A1-FW	AGGACGATGAGGTTCTGCTGGA
mCYP46A1-RV	TCGTAGTCCAGGTGCCTCTT
mCYP11A1-FW	TGCTCAACCTGCCTCCAGACTT
mCYP11A1-RV	ACTGGCTGAAGTCTCGCTTCTG

Table S4. Number of animals used in each read-out performed in this study's trials.

N° Trial	Experimental Groups	Rotarod		Grip Strength		NORT		Mass Spect Analysis (24-OHC)	Mass Spect Analysis (desmosterol, 7-deydrocholesterol)	Gene expression (qPCR)	muHTT aggregates analysis	Plasmatic NfL analysis
		8 weeks	10 weeks	8 weeks	10 weeks	9 weeks	11 weeks	12 weeks	12 weeks	12 weeks	12 weeks	12 weeks
1	WT			6	6	6	6					
	R6/2 - vehicle			3	3	3	3					
	R6/2 - liposomes			3	3	3	2					
2	WT					5	5	4	4			
	R6/2 - vehicle					2	2	5	5			
	R6/2 - liposomes					6	6	7	7			
3	WT	6	6			7	7	8	7			
	R6/2 - vehicle	6	6			6	5	1	5			
	R6/2 - liposomes	6	6			6	6	4	6			
4	WT	9	9					4	4			5
	R6/2 - vehicle	8	8					4	4			4
	R6/2 - liposomes	13	13					4	4			6
5	WT			10	10					3	*	5
	R6/2 - vehicle			9	9					5	3	4
	R6/2 - liposomes			10	10					5	4	6
6	WT							3				
	R6/2 - vehicle							7				
	R6/2 - liposomes							2				
**Total**	**wt**	**15**	**15**	**16**	**16**	**18**	**18**	**19**	**15**	**3**	**0**	**10**
	**R6/2+PBS**	**14**	**14**	**12**	**12**	**11**	**10**	**17**	**14**	**5**	**3**	**8**
	**R6/2+Chol**	**19**	**19**	**13**	**13**	**15**	**14**	**17**	**17**	**5**	**4**	**12**

*The EM48 antibody specifically targets the expanded polyQ tract prone to aggregation; therefore, the analysis was not conducted on wild-type mice, as they do not possess the expanded polyQ tract
